# A New Approach to Entropy and Similarity Measure of Vague Soft Sets

**DOI:** 10.1155/2014/610125

**Published:** 2014-07-24

**Authors:** Dan Hu, Zhiyong Hong, Yong Wang

**Affiliations:** ^1^Xihua University, Chengdu, Sichuan 610039, China; ^2^College of Mathematics, Southwest Jiaotong University, Chengdu, Sichuan 610031, China

## Abstract

We focus our discussion on the uncertainty measures of vague soft sets. We propose axiomatic definitions of similarity measure and entropy for vague soft sets. Furthermore, we present a new category of similarity measures and entropies for vague soft sets. The basic properties of these measures are discussed and the relationships among these measures are analyzed.

## 1. Introduction

In 1999, Molodtsov [1] introduced the concept of soft sets, which can be considered as a new mathematical tool for dealing with uncertainties that traditional mathematical tools cannot handle. A soft set is a collection of approximate descriptions of an object. The absence of restriction on the approximate description in soft set theory makes it very convenient to apply. Recently, applications of soft sets have surged in various areas, including decision making, data analysis, simulation, and texture classification [2–11].

Accordingly, works on soft set theory are progressing rapidly. Maji et al. [12] defined several algebraic operations on soft sets and conducted a theoretical study on the theory of soft sets. Based on [12], Ali et al. [13] introduced some new operations on soft sets and improved the notion of complement of soft set. They proved that certain De Morgan's laws with respect to these new operations hold in soft set theory. Qin and Hong [14] introduced the notion of soft equality and established lattice structures and soft quotient algebras of soft sets. Maji et al. [15] initiated the study on hybrid structures involving soft sets and fuzzy sets. They proposed the notion of fuzzy soft set as a fuzzy generalization of classical soft sets and some basic properties were discussed. Afterwards, many researchers have worked on this concept. Various kinds of extended fuzzy soft sets such as generalized fuzzy soft sets [16], intuitionistic fuzzy soft sets [17, 18], interval-valued fuzzy soft sets [19], vague soft sets [20], interval-valued intuitionistic fuzzy soft sets [21], and soft interval set [22] have been proposed. The combination of soft set and rough set [23] has also been extensively investigated [24–27].

The measurement of uncertainty is an important topic for the theories dealing with uncertainty. Majumdar and Samanta [28] initiated the study of uncertainty measures of soft sets, in which some similarity measures between soft sets were presented. Recently, some related works concerning the uncertainty measures of soft sets, fuzzy soft sets, intuitionistic fuzzy soft set, and vague soft set were presented [29–32]. Wang and Qu [32] introduced axiomatic definitions of entropy, similarity measure, and distance measure for vague soft sets and proposed some formulas to calculate them. This paper is devoted to a further discussion of uncertainty measures for vague soft set. We make an analysis of the uncertainty measures presented in [32] and point out some drawbacks in it. First, a vague soft set is a parameterized family of vague sets on the universe. Different vague soft sets may have different parameter sets. The entropy, similarity measure, and distance measure presented in [32] are actually partial measures in the sense that they take only the vague soft sets with the whole parameter set into account. Second, the axiomatic definition of entropy is not complete; that is, in some cases, the definition cannot guarantee a crisper vague soft set has a smaller entropy. We illustrate this with an example. Based on these observations, we propose a new axiomatic definition of entropy and present a new approach to construct the similarity measures and entropies for vague soft sets. The paper is organized as follows. In Section 2, we recall some notions and properties of soft sets and vague soft sets. In Section 3, we analyze the axiomatic definitions of similarity measure, distance measure, and entropy presented in [32] and point out some drawbacks in it. The new axiomatic definitions of similarity measure and entropy are presented. In Section 4, we propose a new approach to construct similarity measures between vague soft sets.  Section 5 is devoted to the construction of entropy for vague soft set based on similarity measures. The paper is completed with some concluding remarks.

## 2. Overview of Soft Sets and Vague Soft Sets

In this section, we recall some fundamental notions of soft sets and vague soft sets. See especially [1, 20, 33, 34] for further details and background.

The theory of fuzzy sets initiated by Zadeh [34] provides an appropriate framework for representing and processing vague concepts by allowing partial memberships. Let *U* be a nonempty set, called universe. A fuzzy set *μ* on *U* is defined by a membership function *μ* : *U* → [0,1]. For *x* ∈ *U*, the membership value *μ*(*x*) essentially specifies the degree to which *x* belongs to the fuzzy set *μ*. We denote by *F*(*U*) the set of all fuzzy sets on *U*.

Among the extensions of the classic fuzzy set, vague set is one of the most popular sets treating imprecision and uncertainty. It was proposed by Gau and Buehrer [33].

**Definition 1 (see [33]).**

A vague set *A* over the universe *U* can be expressed by the notion *A* = {(*x*, [*t*_*A*_(*x*), 1 − *f*_*A*_(*x*)]); *x* ∈ *U*}; that is, *A*(*x*) = [*t*_*A*_(*x*), 1 − *f*_*A*_(*x*)], and the condition 0 ≤ *t*_*A*_(*x*) ≤ 1 − *f*_*A*_(*x*) should hold for any *x* ∈ *U*, where *t*_*A*_(*x*) is called the membership degree (true membership) of element *x* to the vague set *A*, while *f*_*A*_(*x*) is the degree of nonmembership (false membership) of the element *x* to the vague set *A*.

In this definition, *t*_*A*_(*x*) is a lower bound on the grade of membership of *x* to *A* derived from the evidence for *x* and *f*_*A*_(*x*) is a lower bound on the negation of *x* derived from the evidence against *x*. The vague value [*t*_*A*_(*x*), 1 − *f*_*A*_(*x*)] indicates that the exact grade of membership of *x* to *A* may be unknown, but it is bounded by *t*_*A*_(*x*) and 1 − *f*_*A*_(*x*).

Every fuzzy set *μ* corresponds to the following vague set:

(1) $$\begin{array}{c} \mu =\left\{\left(x,\left[\mu \left(x\right),1-\mu \left(x\right)\right]\right);\;x\in U\right\}. \end{array}$$

Thus, the notion of vague sets is a generalization of fuzzy sets.

**Definition 2 (see [33]).**

Let *A*, *B* be two vague sets over the universe *U*. The union, intersection, and complement of vague sets are defined as follows:

(2) A∪B={(x,[tA(x)∨tB(x),(1−fA(x))∨(1−fB(x))]);  x∈U},A∩B={(x,[tA(x)∧tB(x),(1−fA(x))∧(1−fB(x))]);  x∈U},Ac={(x,[fA(x),1−tA(x)]);  x∈U}.

**Definition 3 (see [33]).**

Let *A*, *B* be two vague sets over the universe *U*. If, for  all  *x* ∈ *U*, *t*_*A*_(*x*) ≤ *t*_*B*_(*x*), 1 − *f*_*A*_(*x*) ≤ 1 − *f*_*B*_(*x*), then *A* is called a vague subset of *B*, denoted by *A*⊆*B*.

The operations on vague sets are natural generalizations of the corresponding operations on fuzzy sets. Also, the notion of vague subset is a generalization of the notion of fuzzy subset.

In 1999, Molodtsov [1] proposed a new concept called soft set to model uncertainties, which associates a set of objects with a set of parameters. Concretely, let *U* be the universe set and *E* the set of all possible parameters under consideration with respect to *U*. Usually, parameters are attributes, characteristics, or properties of objects in *U*. (*U*, *E*) will be called a soft space. Molodtsov defined the notion of a soft set in the following way.

**Definition 4 (see [1]).**

A pair (*F*, *A*) is called a soft set over *U*, where *A*⊆*E* and *F* is a mapping given by *F* : *A* → *P*(*U*).

In other words, a soft set over *U* is a parameterized family of subsets of *U*. *A* is called the parameter set of the soft set (*F*, *A*). For *e* ∈ *A*, *F*(*e*) may be considered as the set of *e*-approximate elements of (*F*, *A*). For illustration, we consider the following example of soft set.

**Example 5 .**

Suppose that there are six houses in the universe *U* given by *U* = {*h*_1_, *h*_2_, *h*_3_, *h*_4_, *h*_5_, *h*_6_} and *E* = {*e*_1_, *e*_2_, *e*_3_, *e*_4_, *e*_5_} is the set of parameters. *e*_1_, *e*_2_, *e*_3_, *e*_4_, and *e*_5_ stand for the parameters “expensive,” “beautiful,” “wooden,” “cheap,” and “in the green surroundings,” respectively.

In this case, to define a soft set means to point out expensive houses, beautiful houses, and so on. The soft set (*F*, *E*) may describe the “attractiveness of the houses” which Mr. X is going to buy. Suppose that *F*(*e*_1_) = {*h*_2_, *h*_4_}, *F*(*e*_2_) = {*h*_1_, *h*_3_}, *F*(*e*_3_) = {*h*_3_, *h*_4_, *h*_5_}, *F*(*e*_4_) = {*h*_1_, *h*_3_, *h*_5_}, and *F*(*e*_5_) = {*h*_1_}. Then the soft set (*F*, *E*) is a parameterized family {*F*(*e*_*i*_); 1 ≤ *i* ≤ 5} of subsets of *U* and gives us a collection of approximate descriptions of an object. *F*(*e*_1_) = {*h*_2_, *h*_4_} means “houses *h*_2_ and *h*_4_” are “expensive.”

Maji et al. [15] initiated the study on hybrid structures involving soft sets and fuzzy sets. They proposed the notion of fuzzy soft set by combining soft sets and fuzzy sets. Afterwards, various kinds of extended fuzzy soft sets were presented. Xu et al. [20] proposed the notion of vague soft set as follows.

**Definition 6 (see [20]).**

Let *U* be an initial universe set, *V*(*U*) the set of all vague sets over *U*, and *E* a set of parameters. A pair (*F*, *A*) is called a vague soft set over *U*, where *A*⊆*E* and *F* is a mapping given by *F* : *A* → *V*(*U*).

In what follows, we denote by VSS(*U*) the set of all vague soft sets over *U*.

**Definition 7 (see [20]).**

For two vague soft sets (*F*, *A*) and (*G*, *B*) over a universe *U*, one says that (*F*, *A*) is a vague soft subset of (*G*, *B*), if *A*⊆*B* and for  all  *e* ∈ *A*, *F*(*e*) is a vague subset of *G*(*e*). This relation is denoted by (*F*, *A*)⊆(*G*, *B*).

**Definition 8 (see [20]).**

A vague soft set (*F*, *A*) over *U* is said to be a null vague soft set, denoted by *∅*_*A*_, if *t*_*F*(*e*)_(*x*) = 0, 1 − *f*_*F*(*e*)_(*x*) = 0 for any *e* ∈ *A* and *x* ∈ *U*.

A vague soft set (*F*, *A*) over *U* is said to be an absolute vague soft set, denoted by *U*_*A*_, if *t*_*F*(*e*)_(*x*) = 1, 1 − *f*_*F*(*e*)_(*x*) = 1 for any *e* ∈ *A* and *x* ∈ *U*.

By the interpretation of true membership and false membership, a null vague soft set *∅*_*A*_ is actually a soft set (*F*, *A*) satisfying *F*(*e*) = *∅* for any *e* ∈ *A* and an absolute vague soft set *U*_*A*_ is actually a soft set (*F*, *A*) satisfying *F*(*e*) = *U* for any *e* ∈ *A*.

**Definition 9 .**

The complement of a vague soft set (*F*, *A*) is denoted by (*F*, *A*)^*c*^ = (*F* ^*c*^, *A*), where *F* ^*c*^ : *A* → *V*(*U*) is a mapping given by *t*_*F*^*c*^(*e*)_(*x*) = *f*_*F*(*e*)_(*x*), 1 − *f*_*F*^*c*^(*e*)_(*x*) = 1 − *t*_*F*(*e*)_(*x*) for any *e* ∈ *A* and *x* ∈ *U*.

**Remark 10 .**

In [32], the complement of a vague soft set (*F*, *A*) is defined by (*F*, *A*)^*c*^ = (*F* ^*c*^, ¬*A*), where ¬*A* = {¬*e*; *e* ∈ *A*}, *F* ^*c*^ : ¬*A* → *V*(*U*), and *t*_*F*^*c*^(¬*e*)_(*x*) = *f*_*F*(*e*)_(*x*), 1 − *f*_*F*^*c*^(¬*e*)_(*x*) = 1 − *t*_*F*(*e*)_(*x*) for any *e* ∈ *A* and *x* ∈ *U*. This definition is in essence equivalent to Definition 9. But by using this definition, it is not convenient when considering the grade of similarity between (*F*, *A*) and (*F*, *A*)^*c*^, because the parameter sets of (*F*, *A*) and (*F*, *A*)^*c*^ are disjoint in mathematics; that is, *A*∩¬*A* = *∅*. In general, comparing approximate sets for different parameters is not reasonable.

## 3. Analysis of the Existing Entropy of Vague Soft Set

The measurement of uncertainty is an important topic for the theory dealing with uncertainty. The entropy, similarity measure, and distance measure in fuzzy set theory and the relationships among these measures have been extensively studied for their wide applications [35–43]. Wang and Qu [32] introduced the concepts of entropy, similarity measure, and distance measure of vague soft sets. Furthermore, the relationships among these measures were analyzed. In this section, we point out some drawbacks in [32] and present new definitions of entropy and similarity measure for vague soft sets.

**Definition 11 (see [32]).**

Let *H* : VSS(*U*)→[0,1] be a mapping. For (*F*, *E*) ∈ VSS(*U*), *H*(*F*, *E*) is called the entropy of (*F*, *E*) if it satisfies the following conditions:

(*H*1) *H*(*F*, *E*) = 0⇔for  all  *e* ∈ *E*, *x* ∈ *U*, *t*_*F*(*e*)_(*x*) = 0, *f*_*F*(*e*)_(*x*) = 1, or *t*_*F*(*e*)_(*x*) = 1, *f*_*F*(*e*)_(*x*) = 0;
(*H*2) *H*(*F*, *E*) = 1⇔for  all  *e* ∈ *E*, *x* ∈ *U*, *t*_*F*(*e*)_(*x*) = *f*_*F*(*e*)_(*x*);
(*H*3) *H*(*F*, *E*) = *H*((*F*, *E*)^*c*^);
(*H*4) for  all  *e* ∈ *E*, *x* ∈ *U*, when (*F*, *E*)⊆(*G*, *E*) and *t*_*G*(*e*)_(*x*) ≤ *f*_*G*(*e*)_(*x*), or (*F*, *E*)⊇(*G*, *E*) and *t*_*G*(*e*)_(*x*) ≥ *f*_*G*(*e*)_(*x*), then *H*(*F*, *E*) ≤ *H*(*G*, *E*).

In this definition, (*H*1) means the entropy of a soft set is minimal. By (*H*2), the entropy of the most vague soft set is maximal. (*H*3) means the entropies of a vague soft set and its complement are equal. (*H*1), (*H*2), and (*H*3) are natural generalizations of the corresponding conditions needed for the entropy of vague set. Now we focus our discussion on the condition (*H*4). If (*F*, *E*)⊆(*G*, *E*), *t*_*G*(*e*)_(*x*) ≤ *f*_*G*(*e*)_(*x*) for any *e* ∈ *E*, *x* ∈ *U*, then *t*_*G*(*e*)_(*x*) ≤ 0.5 by *t*_*G*(*e*)_(*x*) ≤ 1 − *f*_*G*(*e*)_(*x*). In this case, (*F*, *E*)⊆(*G*, *E*) means *F*(*e*) is crisper than *G*(*e*) for any *e* ∈ *E*. Similarly, if (*F*, *E*)⊇(*G*, *E*), *t*_*G*(*e*)_(*x*) ≥ *f*_*G*(*e*)_(*x*) for any *e* ∈ *E*, *x* ∈ *U*, then *F*(*e*) is crisper than *G*(*e*) for any *e* ∈ *E*. Thus (*H*4) is reasonable, but it is not complete. In fact, let (*F*, *E*) be crisper that (*G*, *E*). There may exist parameters *e*_1_, *e*_2_ ∈ *E* such that *F*(*e*_1_)⊆*G*(*e*_1_) and *F*(*e*_2_)⊇*G*(*e*_2_). Also, for a specific parameter *e* ∈ *E*, there may exist some elements *x* ∈ *U*_1_ ⊂ *U* such that *t*_*G*(*e*)_(*x*) ≤ *f*_*G*(*e*)_(*x*), *t*_*F*(*e*)_(*x*) ≤ *t*_*G*(*e*)_(*x*), and *f*_*F*(*e*)_(*x*) ≥ *f*_*G*(*e*)_(*x*), and *t*_*G*(*e*)_(*x*) ≥ *f*_*G*(*e*)_(*x*), *t*_*F*(*e*)_(*x*) ≥ *t*_*G*(*e*)_(*x*), and *f*_*F*(*e*)_(*x*) ≤ *f*_*G*(*e*)_(*x*) for any *x* ∈ *U* − *U*_1_. In these cases, (*H*4) cannot guarantee *H*(*F*, *E*) ≤ *H*(*G*, *E*) because (*F*, *E*)⊈(*G*, *E*) and (*G*, *E*)⊈(*F*, *E*). As illustration, we consider the following example.

**Example 12 .**

(1) Let *E* = {*e*_1_, *e*_2_}, *U* = {*x*_1_}. The vague soft sets (*F*, *E*) and (*G*, *E*) are defined by

(3) $$\begin{array}{c} F\left(e_{1}\right)=\left(x_{1},\left[0.2,0.3\right]\right),\;\;F\left(e_{2}\right)=\left(x_{1},\left[0.7,0.8\right]\right),\\ G\left(e_{1}\right)=\left(x_{1},\left[0.4,0.5\right]\right),\;\;G\left(e_{2}\right)=\left(x_{1},\left[0.6,0.7\right]\right). \end{array}$$

By the interpretation of true membership and false membership, *F*(*e*_1_) and *F*(*e*_2_) are crisper than *G*(*e*_1_) and *G*(*e*_2_), respectively. We noticed that (*F*, *E*)⊈(*G*, *E*) and (*G*, *E*)⊈(*F*, *E*). Thus (*H*4) can not guarantee *H*(*F*, *E*) ≤ *H*(*G*, *E*).

(2) Let *E* = {*e*}, *U* = {*x*_1_, *x*_2_}. The vague soft sets (*F*, *E*) and (*G*, *E*) are defined by

(4) $$\begin{array}{c} F\left(e\right)=\left(x_{1},\left[0.2,0.3\right]\right)+\left(x_{2},\left[0.7,0.8\right]\right),\\ G\left(e\right)=\left(x_{1},\left[0.4,0.5\right]\right)+\left(x_{2},\left[0.6,0.7\right]\right). \end{array}$$

Similarly, *F*(*e*) is crisper than *G*(*e*). Also, (*F*, *E*)⊈(*G*, *E*) and (*G*, *E*)⊈(*F*, *E*). (*H*4) can not guarantee *H*(*F*, *E*) ≤ *H*(*G*, *E*).

By the way, VSS(*U*) is the set of all vague soft sets over *U*, but (*H*1)~(*H*4) are all talking about the vague soft sets with the whole parameter set *E*. Thus, the entropy derived from this definition is actually a partial entropy.

Based on these observations, we introduce the new definition of entropy as follows.

**Definition 13 .**

Let *H* : VSS(*U*)→[0,1] be a mapping. For (*F*, *A*) ∈ VSS(*U*), *H*(*F*, *A*) is called the entropy of (*F*, *A*) if it satisfies the following conditions:

(*H*1) *H*(*F*, *A*) = 0⇔for  all  *e* ∈ *A*, *x* ∈ *U*, *t*_*F*(*e*)_(*x*) = 0, *f*_*F*(*e*)_(*x*) = 1, or *t*_*F*(*e*)_(*x*) = 1, *f*_*F*(*e*)_(*x*) = 0;
(*H*2) *H*(*F*, *A*) = 1⇔for  all  *e* ∈ *A*, *x* ∈ *U*, *t*_*F*(*e*)_(*x*) = *f*_*F*(*e*)_(*x*);
(*H*3) *H*(*F*, *A*) = *H*((*F*, *A*)^*c*^);
(*H*4) Let (*F*, *A*), (*G*, *A*) ∈ VSS(*U*). If for  all  *e* ∈ *A*, *x* ∈ *U*, *t*_*F*(*e*)_(*x*) ≤ *t*_*G*(*e*)_(*x*), *f*_*F*(*e*)_(*x*) ≥ *f*_*G*(*e*)_(*x*) whenever *t*_*G*(*e*)_(*x*) ≤ *f*_*G*(*e*)_(*x*), and *t*_*F*(*e*)_(*x*) ≥ *t*_*G*(*e*)_(*x*), *f*_*F*(*e*)_(*x*) ≤ *f*_*G*(*e*)_(*x*) whenever *t*_*G*(*e*)_(*x*) ≥ *f*_*G*(*e*)_(*x*), then *H*(*F*, *A*) ≤ *H*(*G*, *A*).

We note that the entropy presented in Theorem 3.1 [32] is also an entropy in the sense of Definition 13. Wang and Qu [32] proposed the axioms of similarity measure and distance measure for vague soft sets and discussed the relationships between these measures.

**Definition 14 (Definition 3.2, [32]).**

Let *M* : VSS(*U*) × VSS(*U*)→[0,1] be a mapping. For (*F*, *E*) ∈ VSS(*U*) and (*G*, *E*) ∈ VSS(*U*), *M*((*F*, *E*), (*G*, *E*)) is called the degree of similarity between (*F*, *E*) and (*G*, *E*) if it satisfies the following conditions:

(*M*1)   
*M*((*F*, *E*), (*G*, *E*)) = *M*((*G*, *E*), (*F*, *E*));
(*M*2)   
*M*((*F*, *E*), (*G*, *E*))∈[0,1];
(*M*3)   
*M*((*F*, *E*), (*G*, *E*)) = 1⇔(*F*, *E*) = (*G*, *E*);
(*M*4)   
*M*((*F*, *E*), (*G*, *E*)) = 0⇔for  all  *e* ∈ *E*, *x* ∈ *U*, *t*_*F*(*e*)_(*x*) = 0, *f*_*F*(*e*)_(*x*) = 1, *t*_*G*(*e*)_(*x*) = 1, *f*_*G*(*e*)_(*x*) = 0, or *t*_*F*(*e*)_(*x*) = 1, *f*_*F*(*e*)_(*x*) = 0, *t*_*G*(*e*)_(*x*) = 0, *f*_*G*(*e*)_(*x*) = 1;
(*M*5)   (*F*, *E*)⊆(*G*, *E*)⊆(*P*, *E*) implies *M*((*F*, *E*), (*P*, *E*)) ≤ min⁡(*M*((*F*, *E*), (*G*, *E*)), *M*((*G*, *E*), (*P*, *E*))).

**Definition 15 (Definition 3.2, [32]).**

Let *d* : VSS(*U*) × VSS(*U*)→[0,1] be a mapping. For (*F*, *E*) ∈ VSS(*U*) and (*G*, *E*) ∈ VSS(*U*), *d*((*F*, *E*), (*G*, *E*)) is called the degree of distance between (*F*, *E*) and (*G*, *E*) if it satisfies the following conditions:

(*d*1) *d*((*F*, *E*), (*G*, *E*)) = *d*((*G*, *E*), (*F*, *E*));
(*d*2) *d*((*F*, *E*), (*G*, *E*))∈[0,1];
(*d*3) *d*((*F*, *E*), (*G*, *E*)) = 1⇔for  all  *e* ∈ *E*, *x* ∈ *U*, *t*_*F*(*e*)_(*x*) = 0, *f*_*F*(*e*)_(*x*) = 1, *t*_*G*(*e*)_(*x*) = 1, *f*_*G*(*e*)_(*x*) = 0, or *t*_*F*(*e*)_(*x*) = 1, *f*_*F*(*e*)_(*x*) = 0, *t*_*G*(*e*)_(*x*) = 0, *f*_*G*(*e*)_(*x*) = 1;
(*d*4) *d*((*F*, *E*), (*G*, *E*)) = 0⇔(*F*, *E*) = (*G*, *E*);
(*d*5) (*F*, *E*)⊆(*G*, *E*)⊆(*P*, *E*) implies *d*((*F*, *E*), (*P*, *E*)) ≥ max⁡(*d*((*F*, *E*), (*G*, *E*)), *d*((*G*, *E*), (*P*, *E*))).

**Theorem 16 (see [32]).**

Let *M*((*F*, *E*), (*G*, *E*)) and *d*((*F*, *E*), (*G*, *E*)) be the similarity measure and distance measure between two vague soft sets (*F*, *E*) and (*G*, *E*) as defined in Definitions 13 and 14, respectively. Then the relations between *M*((*F*, *E*), (*G*, *E*)) and *d*((*F*, *E*), (*G*, *E*)) can be given as follows:

(5) $$\begin{array}{c} M\left(\left(F,E\right),\left(G,E\right)\right)+d\left(\left(F,E\right),\left(G,E\right)\right)=1. \end{array}$$

We notice that this theorem holds for the special similarity measure and distance measure presented in Theorems 3.2 and 3.3 [32]. But it does not hold in general; that is, one cannot prove *M*((*F*, *E*), (*G*, *E*)) + *d*((*F*, *E*), (*G*, *E*)) = 1 just by the definitions of similarity measure and distance measure. Furthermore, in Definitions 14 and 15, only the vague soft sets with the whole parameter set *E* are compared. Thus the similarity measure and distance measure are all partial measures. By the way, conditions (*M*2) and (*d*2) are clearly not necessary because the codomain of *M* and *d* has already been restricted to [0,1].

Similarity measure and distance measure are closely related. In what follows, we focus our discussion on entropy and similarity measure. Taking the above observations into account, we propose the following definition of similarity measure for vague soft sets.

**Definition 17 .**

Let *M* : VSS(*U*) × VSS(*U*)→[0,1] be a mapping. For (*F*, *A*) ∈ VSS(*U*) and (*G*, *B*) ∈ VSS(*U*), *M*((*F*, *A*), (*G*, *B*)) is called the degree of similarity between (*F*, *A*) and (*G*, *B*) if it satisfies the following conditions:

(*M*1) *M*((*F*, *A*), (*G*, *B*)) = *M*((*G*, *B*), (*F*, *A*));
(*M*2) *M*((*F*, *A*), (*G*, *B*)) = 1⇔(*F*, *A*) = (*G*, *B*);
(*M*3) *M*((*F*, *A*), (*G*, *B*)) = 0⇔for  all  *e* ∈ *A*∩*B* (*A*∩*B* ≠ *∅*), *x* ∈ *U*, *t*_*F*(*e*)_(*x*) = 0, *f*_*F*(*e*)_(*x*) = 1, *t*_*G*(*e*)_(*x*) = 1, *f*_*G*(*e*)_(*x*) = 0, or *t*_*F*(*e*)_(*x*) = 1, *f*_*F*(*e*)_(*x*) = 0, *t*_*G*(*e*)_(*x*) = 0, *f*_*G*(*e*)_(*x*) = 1;
(*M*4) (*F*, *A*)⊆(*G*, *B*)⊆(*P*, *C*) implies *M*((*F*, *A*), (*P*, *C*)) ≤ min⁡(*M*((*F*, *A*), (*G*, *B*)), *M*((*G*, *B*), (*P*, *C*))).

## 4. Similarity Measures for Vague Soft Sets

Similarity measures quantify the extent to which different patterns, images, or sets are alike. In this section, we propose a new category of similarity measures (in the sense of Definition 17) for vague soft sets.

Based on the notion of fuzzy equivalence proposed by Fodor and Roubens [44], Li et al. [37] proposed an approach to calculate the similarity degree between fuzzy sets. The approach can be summarized as the following theorem. Here some modifications on notations and technical terms have been made to fit the context of our discussion.

**Theorem 18 (see [37]).**

Suppose *N*_*θ*_ and *N*_*δ*_ are functions defined for all *A*, *B* ∈ *F*(*U*) by

(6) Nθ(A,B) =∑x∈U(a−a|A(x)−B(x)| +b·min⁡(A(x),B(x)))  ×(∑x∈U(a−(a−1)|A(x)−B(x)| + b·min⁡(A(x),B(x))))−1,Nδ(A,B) =∑x∈U(a−a|A(x)−B(x)|+b(1−max⁡(A(x),B(x))))  ×(∑x∈U(a−(a−1)|A(x)−B(x)|+ b(1−max⁡(A(x),B(x)))))−1

with *a* ≥ 0, *b* ≥ 0, and *a* + *b* > 0; then, *N*_*θ*_ and *N*_*δ*_ are similarity measures for fuzzy sets in the sense that

1. *N*_*θ*_(*U*, *∅*) = 0, *N*_*δ*_(*U*, *∅*) = 0 and *N*_*θ*_(*A*, *A*) = 1, *N*_*δ*_(*A*, *A*) = 1 whenever *A* ∈ *F*(*U*);
2. *N*_*θ*_(*A*, *B*) = *N*_*θ*_(*B*, *A*), *N*_*δ*_(*A*, *B*) = *N*_*δ*_(*B*, *A*) whenever *A*, *B* ∈ *F*(*U*);
3. for all *A*, *B*, *C* ∈ *F*(*U*), *N*_*θ*_(*A*, *C*) ≤ min⁡(*N*_*θ*_(*A*, *B*), *N*_*θ*_(*B*, *C*)), *N*_*δ*_(*A*, *C*) ≤ min⁡(*N*_*δ*_(*A*, *B*), *N*_*δ*_(*B*, *C*)) whenever *A*⊆*B*⊆*C*.

Here, in order to avoid the denominator being zero, we set 0/0 = 1. By setting particular values of *a* and *b*, one can obtain some typical similarity measures for fuzzy sets [37]. Now we extended these measures to vague soft sets.

**Theorem 19 .**

*M*_*θ*_ : *VSS*(*U*) × *VSS*(*U*) → [0,1] is a similarity measure, where, for any (*F*, *A*), (*G*, *B*) ∈ *VSS*(*U*),

(7) $$\begin{array}{c} M_{\theta }\left(\left(F,A\right),\left(G,B\right)\right)=\frac{1}{\left|A\cup B\right|}\underset{e\in A\cap B}{\sum }N_{\theta }\left(F\left(e\right),G\left(e\right)\right),\\ N_{\theta }\left(F\left(e\right),G\left(e\right)\right)=\frac{1}{2}\left(N_{\theta }\left(t_{F(e)},t_{G(e)}\right)+N_{\theta }\left(f_{F(e)},f_{G(e)}\right)\right). \end{array}$$

**Proof**

(*M*1) is trivial.

(*M*2) If (*F*, *A*) = (*G*, *B*), then *A* = *B* and *t*_*F*(*e*)_ = *t*_*G*(*e*)_, *f*_*F*(*e*)_ = *f*_*G*(*e*)_ for any *e* ∈ *A*. It follows that *N*_*θ*_(*t*_*F*(*e*)_, *t*_*G*(*e*)_) = 1, *N*_*θ*_(*f*_*F*(*e*)_, *f*_*G*(*e*)_) = 1 and hence *N*_*θ*_(*F*(*e*), *G*(*e*)) = 1. Thus *M*_*θ*_((*F*, *A*), (*G*, *B*)) = (1/|*A*|)∑_*e*∈*A*_ *N*_*θ*_(*F*(*e*), *G*(*e*)) = |*A*|/|*A*| = 1.

Conversely, we assume that *M*_*θ*_((*F*, *A*), (*G*, *B*)) = 1. By *N*_*θ*_(*F*(*e*), *G*(*e*)) ≤ 1 we have 1 = *M*_*θ*_((*F*, *A*), (*G*, *B*)) ≤ |*A*∩*B*|/|*A* ∪ *B*|. It follows that *A* = *B* and *N*_*θ*_(*t*_*F*(*e*)_, *t*_*G*(*e*)_) = 1 and *N*_*θ*_(*f*_*F*(*e*)_, *f*_*G*(*e*)_) = 1 for any *e* ∈ *A*. If there exist *e* ∈ *A*, *x* ∈ *U* such that *t*_*F*(*e*)_(*x*) ≠ *t*_*G*(*e*)_(*x*), then

(8) ∑x∈U(a−(a−1)|tF(e)(x)−tG(e)(x)|+ b·min⁡(tF(e)(x),tF(e)(x))) =∑x∈U(a−a|tF(e)(x)−tG(e)(x)|+ b·min⁡(tF(e)(x),tF(e)(x)))  +∑x∈U|tF(e)(x)−tG(e)(x)| >∑x∈U(a−a|tF(e)(x)−tG(e)(x)|+ b·min⁡(tF(e)(x),tF(e)(x)))

and consequently, *N*_*θ*_(*t*_*F*(*e*)_, *t*_*G*(*e*)_) < 1. This is a contradiction. Thus *t*_*F*(*e*)_(*x*) = *t*_*G*(*e*)_(*x*) for each *e* ∈ *A*, *x* ∈ *U*. Similarly, we have *f*_*F*(*e*)_(*x*) = *f*_*G*(*e*)_(*x*) for each *e* ∈ *A*, *x* ∈ *U*. Consequently, we have (*F*, *A*) = (*G*, *B*).

(*M*3) Let *M*((*F*, *A*), (*G*, *B*)) = 0. It follows that, for each *e* ∈ *A*∩*B*, *N*_*θ*_(*F*(*e*), *G*(*e*)) = 0 and hence *N*_*θ*_(*t*_*F*(*e*)_, *t*_*G*(*e*)_) = 0, *N*_*θ*_(*f*_*F*(*e*)_, *f*_*G*(*e*)_) = 0. By *N*_*θ*_(*t*_*F*(*e*)_, *t*_*G*(*e*)_) = 0 we have, for each *x* ∈ *U*, *t*_*F*(*e*)_(*x*) = 0, *t*_*G*(*e*)_(*x*) = 1 or *t*_*F*(*e*)_(*x*) = 1, *t*_*G*(*e*)_(*x*) = 0.

1. If *t*_*F*(*e*)_(*x*) = 0, *t*_*G*(*e*)_(*x*) = 1, then *f*_*G*(*e*)_(*x*) = 0 by *t*_*G*(*e*)_(*x*) + *f*_*G*(*e*)_(*x*) ≤ 1. Thus *t*_*G*(*e*)_(*x*) = 1 by *N*_*θ*_(*f*_*F*(*e*)_, *f*_*G*(*e*)_) = 0.
2. If *t*_*F*(*e*)_(*x*) = 1, *t*_*G*(*e*)_(*x*) = 0, then *f*_*F*(*e*)_(*x*) = 0 by *t*_*F*(*e*)_(*x*) + *f*_*F*(*e*)_(*x*) ≤ 1. Therefore *f*_*G*(*e*)_(*x*) = 1 by *N*_*θ*_(*f*_*F*(*e*)_, *f*_*G*(*e*)_) = 0.

The converse implication is trivial.

(*M*4) Let (*F*, *A*), (*G*, *B*), (*P*, *C*) ∈ VSS(*U*) and (*F*, *A*)⊆(*G*, *B*)⊆(*P*, *C*). It follows that *A*⊆*B*⊆*C* and *F*(*e*)⊆*G*(*e*)⊆*P*(*e*) for each *e* ∈ *A*. Consequently, *t*_*F*(*e*)_⊆*t*_*G*(*e*)_⊆*t*_*P*(*e*)_, *f*_*P*(*e*)_⊆*f*_*G*(*e*)_⊆*f*_*F*(*e*)_. Thus we have *N*_*θ*_(*t*_*F*(*e*)_, *t*_*P*(*e*)_) ≤ *N*_*θ*_(*t*_*F*(*e*)_, *t*_*G*(*e*)_), *N*_*θ*_(*t*_*F*(*e*)_, *t*_*P*(*e*)_) ≤ *N*_*θ*_(*t*_*G*(*e*)_, *t*_*P*(*e*)_), *N*_*θ*_(*f*_*F*(*e*)_, *f*_*P*(*e*)_) ≤ *N*_*θ*_(*f*_*F*(*e*)_, *f*_*G*(*e*)_), *N*_*θ*_(*f*_*F*(*e*)_, *f*_*P*(*e*)_) ≤ *N*_*θ*_(*f*_*G*(*e*)_, *f*_*P*(*e*)_), and hence *N*_*θ*_(*F*(*e*), *P*(*e*)) ≤ *N*_*θ*_(*F*(*e*), *G*(*e*)), *N*_*θ*_(*F*(*e*), *P*(*e*)) ≤ *N*_*θ*_(*G*(*e*), *P*(*e*)). Therefore we obtain

(9) Sθ((F,A),(G,B))=1|B|∑e∈ANθ(F(e),G(e))≥1|C|∑e∈ANθ(F(e),P(e))=Sθ((F,A),(P,C)),Sθ((G,B),(P,C))=1|C|∑e∈BNθ(G(e),P(e))≥1|C|∑e∈ANθ(G(e),P(e))≥1|C|∑e∈ANθ(F(e),P(e))=Sθ((F,A),(P,C)).

This completes the proof.

This theorem presents a category of similarity measures for vague soft sets. In (7), let *a* = 0, *b* = 1; then, we have

(10) Nθ(tF(e),tG(e)) =∑x∈Umin⁡(tF(e)(x),tG(e)(x))  ×(∑x∈U(|tF(e)(x)−tG(e)(x)|       +min⁡(tF(e)(x),tG(e)(x))))−1 =∑x∈Umin⁡⁡(tF(e)(x),tG(e)(x))∑x∈Umax⁡⁡(tF(e)(x),tG(e)(x)),Nθ(fF(e),fG(e)) =∑x∈Umin⁡⁡(fF(e)(x),fG(e)(x))∑x∈Umax⁡⁡(fF(e)(x),fG(e)(x)).

Therefore we obtain

(11) M1((F,A),(G,B))=12|A∪B|∑e∈A∩B(∑x∈Umin⁡(tF(e)(x),tG(e)(x))∑x∈Umax⁡(tF(e)(x),tG(e)(x))+∑x∈Umin⁡⁡(fF(e)(x),fG(e)(x))∑x∈Umax⁡⁡(fF(e)(x),fG(e)(x))).

Similarly, let *a* = 0, *b* = 2; then, we have

(12) M2((F,A),(G,B)) =1|A∪B|∑e∈A∩B(∑x∈Umin⁡(tF(e)(x),tG(e)(x))∑x∈U(tF(e)(xi)+tG(e)(xi)) +∑x∈Umin⁡(fF(e)(x),fG(e)(x))∑x∈U(fF(e)(xi)+fG(e)(xi))).

Let *a* = 0.5, *b* = 0; then, we have

(13) M3((F,A),(G,B)) =12|A∪B|∑e∈A∩B(∑x∈U(1−|tF(e)(x)−tG(e)(x)|)∑x∈U(1+|tF(e)(x)−tG(e)(x)|) +∑x∈U(1−|fF(e)(x)−fG(e)(x)|)∑x∈U(1+|fF(e)(x)−fG(e)(x)|)).

Let *a* = 1, *b* = 0; then, we have

(14) M4((F,A),(G,B)) =1|A∪B|∑e∈A∩B(1−12|U| ×∑x∈U(|tF(e)(x)−tG(e)(x)|‍ +|fF(e)(x)−fG(e)(x)|)).

Let *a* = 2, *b* = 0; then, we have

(15) M5((F,A),(G,B)) =1|A∪B|∑e∈A∩B(∑x∈U(1−|tF(e)(x)−tG(e)(x)|)∑x∈U(2−|tF(e)(x)−tG(e)(x)|)+∑x∈U(1−|fF(e)(x)−fG(e)(x)|)∑x∈U(2−|fF(e)(x)−fG(e)(x)|)).

Let *a* = 1, *b* = 1; then, we have

(16) M6((F,A),(G,B)) =12|A∪B|  ×∑e∈A∩B(2−∑x∈U|tF(e)(x)−tG(e)(x)|∑x∈U(1+min⁡(tF(e)(x),tG(e)(x)))       −∑x∈U|fF(e)(x)−fG(e)(x)|∑x∈U(1+min⁡(fF(e)(x),fG(e)(x)))).

**Theorem 20 .**

*M*_*δ*_ : *VSS*(*U*) × *VSS*(*U*)→[0,1] is a similarity measure, where, for any (*F*, *A*), (*G*, *B*) ∈ *VSS*(*U*),

(17) $$\begin{array}{c} M_{\delta }\left(\left(F,A\right),\left(G,B\right)\right)=\frac{1}{\left|A\cup B\right|}\underset{e\in A\cap B}{\sum }N_{\delta }\left(F\left(e\right),G\left(e\right)\right),\\ N_{\delta }\left(F\left(e\right),G\left(e\right)\right)=\frac{1}{2}\left(N_{\delta }\left(t_{F(e)},t_{G(e)}\right)+N_{\delta }\left(f_{F(e)},f_{G(e)}\right)\right). \end{array}$$

**Proof**

It can be proved in the same manner with Theorem 19.

In (17), let *a* = 0, *b* = 1; then, we have

(18) M7((F,A),(G,B))=12|A∪B| ×∑e∈A∩B(∑x∈U(1−max⁡(tF(e)(x),tG(e)(x)))∑x∈U(1−min⁡(tF(e)(x),tG(e)(x)))+∑x∈U(1−max⁡(fF(e)(x),fG(e)(x)))∑x∈U(1−min⁡(fF(e)(x),fG(e)(x)))).

Let *a* = 0, *b* = 2; then, we have

(19) M8((F,A),(G,B))=1|A∪B| ×∑e∈A∩B(∑x∈U(1−max⁡(tF(e)(x),tG(e)(x)))∑x∈U(2−tF(e)(x)−tG(e)(x))+∑x∈U(1−max⁡(fF(e)(x),fG(e)(x)))∑x∈U(2−fF(e)(x)−fG(e)(x))).

**Example 21 .**

Let *U* = {*x*_1_, *x*_2_}, *E* = {*e*_1_, *e*_2_, *e*_3_, *e*_4_}, *A* = {*e*_1_, *e*_2_, *e*_3_}, and *B* = {*e*_1_, *e*_2_, *e*_4_}. Suppose that (*F*, *A*) and (*G*, *B*) are vague soft sets over *U* given by

(20) F(e1)=[0,0.5]x1+[0.2,0.4]x2,  G(e1)=[0.3,0.6]x1+[0.4,0.5]x2,F(e2)=[0.5,0.5]x1+[0.5,0.5]x2,G(e2)=[0.5,0.5]x1+[0.4,0.7]x2,F(e3)=[0,0]x1+[1,1]x2,G(e4)=[0,0]x1+[1,1]x2.

By the definition, we have

(21) ∑x∈Umin⁡(tF(e1)(x),tG(e1)(x))∑x∈Umax⁡(tF(e1)(x),tG(e1)(x))=0+0.20.3+0.4=27,∑x∈Umin⁡(fF(e1)(x),fG(e1)(x))∑x∈Umax⁡(fF(e1)(x),fG(e1)(x))=0.4+0.50.5+0.6=911,∑x∈Umin⁡(tF(e2)(x),tG(e2)(x))∑x∈Umax⁡(tF(e2)(x),tG(e2)(x))=0.5+0.40.5+0.5=910,∑x∈Umin⁡(fF(e2)(x),fG(e2)(x))∑x∈Umax⁡(fF(e2)(x),fG(e2)(x))=0.5+0.30.5+0.5=810.

It follows that

(22) $$\begin{array}{c} M_{1}\left(\left(F,A\right),\left(G,B\right)\right)=\frac{1}{8}\left(\frac{2}{7}+\frac{9}{11}+\frac{9}{10}+\frac{8}{10}\right)\approx 0.35. \end{array}$$

Similarly, we have

(23) $$\begin{array}{c} M_{2}\left(\left(F,A\right),\left(G,B\right)\right)\approx 0.4,\\ M_{3}\left(\left(F,A\right),\left(G,B\right)\right)\approx 0.39,\\ M_{4}\left(\left(F,A\right),\left(G,B\right)\right)\approx 0.44,\\ M_{5}\left(\left(F,A\right),\left(G,B\right)\right)\approx 0.47,\\ M_{6}\left(\left(F,A\right),\left(G,B\right)\right)\approx 0.45,\\ M_{7}\left(\left(F,A\right),\left(G,B\right)\right)\approx 0.41,\\ M_{8}\left(\left(F,A\right),\left(G,B\right)\right)\approx 0.45. \end{array}$$

Let *A*, *B* ∈ *F*(*U*). We consider *N*_*θ*_(*A*, *B*). For any *x* ∈ *U*, by 1 − |*A*(*x*) − *B*(*x*)| ≥ 0, min⁡(*A*(*x*), *B*(*x*)) ≥ 0, *a* − *a*|*A*(*x*) − *B*(*x*)| + *b* · min⁡(*A*(*x*), *B*(*x*)) = *a*(1 − |*A*(*x*) − *B*(*x*)|) + *b* · min⁡(*A*(*x*), *B*(*x*)) we conclude that ∑_*x*∈*U*_(*a* − *a* | *A*(*x*) − *B*(*x*)|+*b* · min⁡(*A*(*x*), *B*(*x*))) is increasing with respect to *a* and *b*. Therefore

(24) ∑x∈U|A(x)−B(x)|∑x∈U(a−a|A(x)−B(x)|+b·min⁡(A(x),B(x)))

is decreasing with respect to *a* and *b*. By

(25) Nθ(A,B)=1(1+(∑x∈U|A(x)−B(x)|     ×∑x∈U(a−a|A(x)−B(x)|      +b·min⁡(A(x),B(x))))−1)−1,

it follows that *N*_*θ*_(*A*, *B*) is increasing with respect to *a* and *b*. Thus we have the following corollary.

**Corollary 22 .**

Let (*F*, *A*), (*G*, *B*) ∈ *VSS*(*U*). Then

1. *M*_1_((*F*, *A*), (*G*, *B*)) ≤ *M*_2_((*F*, *A*), (*G*, *B*)), *M*_1_((*F*, *A*), (*G*, *B*)) ≤ *M*_6_((*F*, *A*), (*G*, *B*));
2. *M*_3_((*F*, *A*), (*G*, *B*)) ≤ *M*_4_((*F*, *A*), (*G*, *B*)) ≤ *M*_5_((*F*, *A*), (*G*, *B*));
3. (*M*_4_((*F*, *A*), (*G*, *B*)) ≤ *M*_6_((*F*, *A*), (*G*, *B*)).

This corollary shows the relationships among the similarity measures presented in this section. Similarly, we have the following.

**Corollary 23 .**

Let (*F*, *A*), (*G*, *B*) ∈ *VSS*(*U*). Then *M*_7_((*F*, *A*), (*G*, *B*)) ≤ *M*_8_((*F*, *A*), (*G*, *B*)).

## 5. Entropy Measures for Fuzzy Soft Sets

In this section, we present some entropies for vague soft sets in the sense of Definition 13.

**Theorem 24 .**

*H*_*θ*_(*F*, *A*) is an entropy, where

(26) Hθ(F,A)=Mθ((F,A),(Fc,A))

for any (*F*, *A*) ∈ *VSS*(*U*).

**Proof**

(*H*1) We note that *M*_*θ*_ is a similarity measure. For any (*F*, *A*) ∈ VSS(*U*), we have *H*_*θ*_(*F*, *A*) = 0⇔*M*_*θ*_((*F*, *A*), (*F* ^*c*^, *A*)) = 0⇔for  all  *e* ∈ *A*, *x* ∈ *U*, *t*_*F*(*e*)_(*x*) = 0, *f*_*F*(*e*)_(*x*) = 1, *t*_*F*^*c*^(*e*)_(*x*) = 1, *f*_*F*^*C*^(*e*)_(*x*) = 0, or *t*_*F*(*e*)_(*x*) = 1, *f*_*F*(*e*)_(*x*) = 0, *t*_*F*^*c*^(*e*)_(*x*) = 0, *f*_*F*^*c*^(*e*)_(*x*) = 1⇔for  all  *e* ∈ *A*, *x* ∈ *U*, *t*_*F*(*e*)_(*x*) = 0, *f*_*F*(*e*)_(*x*) = 1, or *t*_*F*(*e*)_(*x*) = 1, *f*_*F*(*e*)_(*x*) = 0.

(*H*2) For any (*F*, *A*) ∈ VSS(*U*), we have *H*_*θ*_(*F*, *A*) = 1⇔*M*_*θ*_((*F*, *A*), (*F* ^*c*^, *A*)) = 1⇔(*F*, *A*) = (*F* ^*c*^, *A*)⇔for  all  *e* ∈ *A*, *x* ∈ *U*, *t*_*F*(*e*)_(*x*) = *t*_*F*^*c*^(*e*)_(*x*), *f*_*F*(*e*)_(*x*) = *f*_*F*^*c*^(*e*)_(*x*)⇔for  all  *e* ∈ *A*, *x* ∈ *U*, *t*_*F*(*e*)_(*x*) = *f*_*F*(*e*)_(*x*).

(*H*3) is trivial.

(*H*4) Let (*F*, *A*), (*G*, *A*) ∈ VSS(*U*), and for  all  *e* ∈ *A*, *x* ∈ *U*, *t*_*F*(*e*)_(*x*) ≤ *t*_*G*(*e*)_(*x*), *f*_*F*(*e*)_(*x*) ≥ *f*_*G*(*e*)_(*x*) if *t*_*G*(*e*)_(*x*) ≤ *f*_*G*(*e*)_(*x*), and *t*_*F*(*e*)_(*x*) ≥ *t*_*G*(*e*)_(*x*), *f*_*F*(*e*)_(*x*) ≤ *f*_*G*(*e*)_(*x*) if *t*_*G*(*e*)_(*x*) ≥ *f*_*G*(*e*)_(*x*). We note that *N*_*θ*_(*t*_*F*(*e*)_, *t*_*F*^*c*^(*e*)_) = *N*_*θ*_(*f*_*F*(*e*)_, *f*_*F*^*c*^(*e*)_) = *N*_*θ*_(*t*_*F*(*e*)_, *f*_*F*(*e*)_) and hence *H*_*θ*_(*F*, *A*) = (1/|*A*|)∑_*e*∈*A*_ *N*_*θ*_(*t*_*F*(*e*)_, *f*_*F*(*e*)_).

By the definition, for each *e* ∈ *A*, we have

(27) Nθ(tF(e),fF(e)) =∑x∈U(a−a|tF(e)(x)−fF(e)(x)|+b·min⁡(tF(e)(x),fF(e)(x)))  ×(∑x∈U(a−(a−1)|tF(e)(x)−fF(e)(x)|+ b·min⁡(tF(e)(x),fF(e)(x))))−1,Nθ(tG(e),fG(e)) =∑x∈U(a−a|tG(e)(x)−fG(e)(x)|+ b·min⁡⁡(tG(e)(x),fG(e)(x)))  ×(∑x∈U(a−(a−1)|tG(e)(x)−fG(e)(x)|+ b·min⁡⁡(tG(e)(x),fG(e)(x))))−1.

Let *U* = {*x*_1_, *x*_2_,…, *x*_*n*_}. Considering the following functions:

(28) g(y1,z1,…,yn,zn) =∑i=1n(a−a|yi−zi|+b·min⁡(yi,zi)),h(y1,z1,…,yn,zn) =∑i=1n(a−(a−1)|yi−zi|+b·min⁡(yi,zi)),f(y1,z1,…,yn,zn)=g(y1,z1,…,yn,zn)h(y1,z1,…,yn,zn),

where *y*_*i*_, *z*_*i*_ ∈ [0,1], *y*_*i*_ + *z*_*i*_ ≤ 1. If *y*_*j*_ ≤ *z*_*j*_, then we have

(29) ∂f∂yj =b∑i=1n|yi−zi|+∑i=1n(a+bmin⁡(yi,zi))h2(y1,z1,…,yn,zn)≥0,∂f∂zj =(−a)∑i=1n|yi−zi|−g(y1,z1,…,yn,zn)h2(y1,z1,…,yn,zn)≤0.

Therefore, we can conclude that *f* is increasing with respect to *y*_*j*_ and decreasing with respect to *z*_*j*_ if *y*_*j*_ ≤ *z*_*j*_. Similarly, *f* is decreasing with respect to *y*_*j*_ and increasing with respect to *z*_*j*_ if *y*_*j*_ ≥ *z*_*j*_. It follows that *N*_*θ*_(*t*_*F*(*e*)_, *f*_*F*(*e*)_) ≤ *N*_*θ*_(*t*_*G*(*e*)_, *f*_*G*(*e*)_) and consequently, *H*_*θ*_(*F*, *A*) ≤ *H*_*θ*_(*G*, *A*).

**Theorem 25 .**

*H*_*δ*_(*F*, *A*) is an entropy, where

(30) $$\begin{array}{c} H_{\delta }\left(F,A\right)=M_{\delta }\left(\left(F,A\right),\left(F^{c},A\right)\right) \end{array}$$

for any (*F*, *A*) ∈ *VSS*(*U*).

**Proof**

The proof is similar to that of Theorem 24.

Using similarity measures *M*_*i*_  (1 ≤ *i* ≤ 8), we can obtain the corresponding entropies *H*_*i*_  (1 ≤ *i* ≤ 8) as follows:

(31) H1(F,A)=1|A|∑e∈A∑x∈Umin⁡⁡(tF(e)(x),fF(e)(x))∑x∈Umax⁡⁡(tF(e)(x),fF(e)(x)),H2(F,A)=2|A|∑e∈A∑x∈Umin⁡⁡(tF(e)(x),fF(e)(x))∑x∈U(tF(e)(x)+fF(e)(x)),H3(F,A)=1|A|∑e∈A∑x∈U(1−|tF(e)(x)−fF(e)(x)|)∑x∈U(1+|tF(e)(x)−fF(e)(x)|),H4(F,A)=1|A|∑e∈A(1−1|U|∑x∈U|tF(e)(x)−fF(e)(x)|),H5(F,A)=2|A|∑e∈A∑x∈U(1−|tF(e)(x)−fF(e)(x)|)∑x∈U(2−|tF(e)(x)−fF(e)(x)|),H6(F,A) =1|A|∑e∈A(1−∑x∈U|tF(e)(x)−fF(e)(x)|∑x∈U(1+min⁡⁡(tF(e)(x),fF(e)(x)))),H7(F,A)=1|A|∑e∈A∑x∈U(1−max⁡(tF(e)(x),fF(e)(x)))∑x∈U(1−min⁡(tF(e)(x),fF(e)(x))),H8(F,A)=2|A|∑x∈U(1−max⁡(tF(e)(x),fF(e)(x)))∑x∈U(2−tF(e)(x)−fF(e)(x)).

**Example 26 .**

We consider the vague soft set (*F*, *A*) given in Example 21

(32) H1(F,A)=13(0+0.20.5+0.6+0.5+0.50.5+0.5+0+01+1)≈0.39,H2(F,A)≈0.44,  H3(F,A)≈0.46,H4(F,A)≈0.52,  H5(F,A)≈0.57,H6(F,A)≈0.53,  H7(F,A)=0.5,  H8(F,A)≈0.56.

## 6. Concluding Remarks

Soft set theory was originally proposed as a general mathematical tool for dealing with uncertainties. Wang and Qu [32] introduced axiomatic definitions of entropy, similarity measure, and distance measure for vague soft sets and proposed some formulas to calculate them. This paper is devoted to a further discussion along this line. We point out that there are some drawbacks in [32] by examples. We propose a new axiomatic definition of entropy and present a new approach to construct the similarity measures and entropies for vague soft sets. Based on these uncertainty measures, we can further probe the applications of vague soft sets in the fields such as pattern recognition, data analysis, and decision making.
